# Antitumor activity of sulfated hyaluronic acid fragments in pre-clinical models of bladder cancer

**DOI:** 10.18632/oncotarget.10529

**Published:** 2016-07-11

**Authors:** Andre R. Jordan, Soum D. Lokeshwar, Luis E. Lopez, Martin Hennig, Juan Chipollini, Travis Yates, Marie C. Hupe, Axel S. Merseburger, Aviva Shiedlin, Wolfgang H. Cerwinka, Kebin Liu, Vinata B. Lokeshwar

**Affiliations:** ^1^ Sheila and David Fuente Graduate Program in Cancer Biology, Sylvester Comprehensive Cancer Center, University of Miami-Miller School of Medicine, Miami, FL, USA; ^2^ Honors Program in Medical Education, University of Miami-Miller School of Medicine, Miami, FL, USA; ^3^ Department of Biochemistry and Molecular Biology, Medical College of Georgia, Augusta University, Augusta, GA, USA; ^4^ Department of Urology, University of Lübeck, Lübeck, Germany; ^5^ Department of Urology, University of Miami-Miller School of Medicine, Miami, FL, USA; ^6^ Drug and Biomaterial R&D, Genzyme Corporation, Cambridge, MA, USA

**Keywords:** bladder cancer, hyaluronic acid, hyaluronidase, sulfated-HA, targeted therapy

## Abstract

Tumor cell-derived hyaluronidase HYAL-1 degrades hyaluronic acid (HA) into angiogenic fragments (AGF: 10-12 disaccharides). AGF support tumor growth and progression. Urine and tissue HAase/HYAL-1 levels are sensitive markers for high-grade bladder cancer (BCa) and its metastasis. In preclinical models of BCa, we evaluated whether o-sulfated AGF (sHA-F) inhibits HAase activity and has antitumor activity. At IC_50_ for HAase activity inhibition (5-20 μg/ml [0.4-1.7 μM]), sHA-F significantly inhibited proliferation, motility and invasion of HYAL-1 expressing BCa cells (253J-Lung, HT1376, UMUC-3), P<0.001. sHA-F did not affect the growth of HYAL-1 non-expressing BCa (5637, RT4, T24, TCCSUP) and normal urothelial (Urotsa, SV-HUC1) cells. sHA-F treatment induced apoptosis by death receptor pathway. sHA-F downregulated transcript and/or protein levels of HA receptors (CD44, RHAMM), p-AKT, β-catenin, pβ-Catenin(S552), Snail and Twist but increased levels of pβ-Catenin(T41/S45), pGSK-3α/β(S21/S9) and E-cadherin. sHA-F also inhibited CD44/Phosphoinositide 3-kinase (PI-3K) complex formation and PI-3K activity. AGF addition or myristoylated-AKT overexpression attenuated sHA-F effects. Contrarily, HYAL-1 expression sensitized RT4 cells to sHA-F treatment. In the 253J-L and HT1376 xenograft models, sHA-F treatment significantly inhibited tumor growth (P<0.001), plausibly by inhibiting angiogenesis and HA receptor-PI-3K/AKT signaling. This study delineates that sHA-F targets tumor-associated HA-HAase system and could be potentially useful in BCa treatment.

## INTRODUCTION

Frequent recurrence and tumor heterogeneity in terms of metastasis make bladder cancer (BCa) one of the costliest cancers to treat and manage clinically. The relative ease of developing urine-based tests has put BCa at the forefront of new biomarker tests; however, targeted treatments to effectively manage and treat advanced BCa are needed [1]. If biomarkers of advanced BCa also function as molecular determinants of tumor growth, invasion and angiogenesis, treatments targeting these biomarkers should be effective in controlling BCa. HAases are a family of enzymes that degrade hyaluronic acid (HA), a non-sulfated glycosaminoglycan. While HA is synthesized by tumor-associated stroma and tumor cells, HAase is expressed exclusively by tumor cells [2, 3]. Furthermore, at present HYAL-1 is the only tumor-derived HAase that has been identified [4]. In tumor tissues, HYAL-1 degrades tumor-associated HA into fragments, some of which are angiogenic. Such angiogenic fragments (10-15 disaccharide units) have been found in the urine of high-grade BCa patients [5, 6]. Work published from our laboratory in bladder and prostate cancer systems, and which was later confirmed by other laboratories, has shown that HYAL-1 and the tumor-associated HA-HAase system promote tumor growth, invasion/metastasis and angiogenesis [7–10]. This suggests that the HA-HYAL-1 system can be targeted for cancer therapy.

Initial published studies from our laboratory involving over 500 patients demonstrated that urinary HAase levels, measured in an activity assay (HAase test), were elevated in high-grade BCa patients and the combined HA and HAase levels have over 80% accuracy in diagnosing BCa and for monitoring its recurrence [11–13]. We have also demonstrated that HYAL-1 mRNA levels expressed in exfoliated urothelial cells that are shed in the urine of BCa patients are an accurate marker for diagnosing BCa [14]. Furthermore, patients with a history of BCa who have elevated HYAL-1 mRNA or urinary HAase levels are at an increased risk for BCa recurrence (i.e. emergence of a new tumor in the bladder) within six months [11–14]. HYAL-1 mRNA levels and protein expression in BCa tissues are also independent and accurate predictors of metastasis and disease-specific mortality in BCa patients [14, 15]. Although HYAL-1 expression has been shown to be decreased in a few cancer types, in several cancer systems, levels of HYAL-1 and other associated HA-family of molecules, as well as presence of low molecular mass HA fragments generated by HYAL-1, have been shown to correlate with disease progression [16–25].

We recently demonstrated that a small molecule inhibitor of HA synthesis, 4-methylumbelliferone, has good efficacy in the chemoprevention and treatment of prostate cancer [26, 27]. Targeting of HA-synthesis by 4-methylumbelliferone for therapy has also been achieved in other cancer systems [28–31]. However, except for our previous study on prostate cancer models, HYAL-1 and/or HAase activity have not been targeted in other preclinical models. We have previously shown that sulfated HA (sHA), generated by o-sulfation of the large HA polymer, inhibits HYAL-1 activity through a mixed inhibition mechanism; sHA is 15-fold better as an uncompetitive inhibitor of HYAL-1 than as a competitive inhibitor [32]. sHA2.75, which has an average molecular mass of 320-490 kDa inhibited prostate cancer cell proliferation, motility, and invasion *in vitro*, and abrogated tumor growth in xenograft models [10]. Since HYAL-1 is an accurate prognostic marker of BCa metastasis and promotes BCa growth and progression, we evaluated whether sulfation of angiogenic HA fragments (AGF), which are the products of HYAL-1 mediated HA degradation, can inhibit HAase activity and display targeted antitumor activity in BCa models. We also investigated the mechanism of action of sulfated HA-fragments (sHA-F).

## RESULTS

### sHA-F inhibited HAase activity and proliferation of HYAL-1 expressing BCa cells

**Figure 1:**
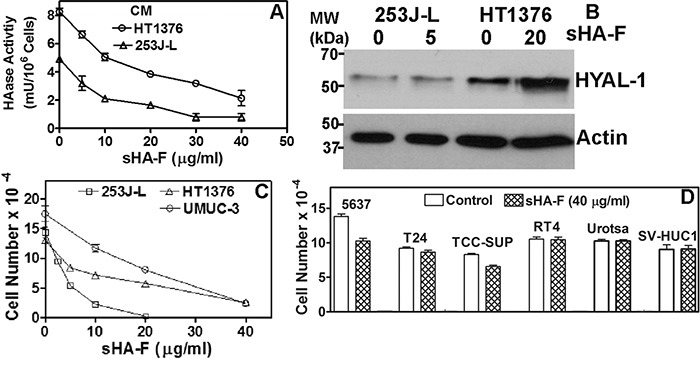
Effect of sHA-F on HAase activity and cell proliferation **A**. Effect of sHA-F on HAase activity (mU/10^6^ cells) in a HAase ELISA like assay (duplicate measurement for each concentration). **B**. Immunoblot analysis of serum-free conditioned media of 253J-L and HT1376 cells collected after 48-hour treatment with sHA-F. **C** and **D**. BCa cells were treated with sHA-F for 72 hours and viable cells were counted. Data: Mean ± sd (quadruplicate). Figure C, the differences in cell numbers between untreated control and sHA-F treated samples were significant (P ≤ 0.002 at each sHA-F concentration). Figure D: Control versus sHA-F treatment; P > 0.05 for each cell line.

We have previously shown that HYAL-1 is the tumor-derived HAase expressed in BCa cells and it is secreted in the conditioned media [4]. Among the BCa cells examined, detectable levels of HAase activity (mU/mg protein) were present in the conditioned media of HT1376 (46±6.7), 253J-Lung (23.5±6.1) and UMUC-3 (6.0±1.8) cells (Supplement Figure 1A). sHA-F inhibited HAase activity present in the conditioned media of both HT1376 and 253J-L cells in a dose-dependent manner; IC_50_∼ 5-20-μg/ml or 0.4 – 0.8 μM (Figure 1A). However, sHA-F treatment did not decrease HYAL-1 protein levels in the conditioned media, or HYAL-1 transcript levels (Figure 1B, Supplemental Figure 1B). This shows that sHA-F inhibits HAase activity and not the HYAL-1 expression. As shown in Figure 1C, sHA-F inhibited the growth of all three HYAL-1 expressing cell lines in a dose-dependent manner; IC_50_ for growth inhibition were 5-μg/ml for 253J-Lung and 20-μg/ml for HT1376 and UMUC-3 cells. At concentrations ≥ IC_50_, the differences in cell numbers between untreated and sHA-F treated samples were statistically significant (P ≤ 0.002; unpaired t-test). HYAL-1 non-expressing BCa cells and also normal urothelial cells (Urotsa, SV-HUC1) were resistant to growth inhibition by sHA-F (Figure 1D). Growth of both 253J-L and HT1376 cells was inhibited in a dose-dependent manner by sHA2k (i.e., sulfated HA-fragments of average size 2,000 Da; Supplement Figure 1C). This suggested that even the smallest products of HA degradation by HYAL-1 (3 – 4 disaccharide units) when sulfated effectively inhibit the growth of HYAL-1 expressing BCa cells.

**Figure 2:**
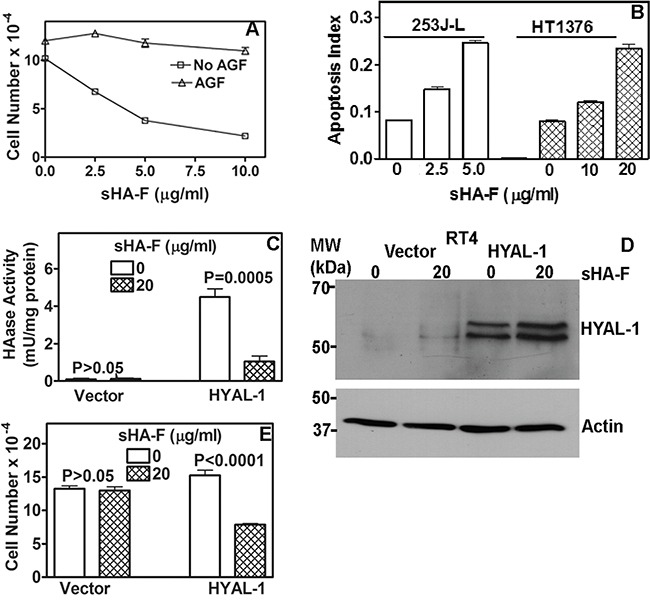
Effect of sHA-F on HAase activity, growth and apoptosis and its attenuation by AGF **A**. 253J-L cells were treated with sHA-F in the presence or absence of AGF (50-μg/ml) for 72 hours and viable cells were counted. Data: Mean ± sd (quadruplicate). P < 0.001; between AGF treated and untreated samples at each sHA-F concentration. **B**. Measurement of apoptosis in cells treated with sHA-F for 48 hours. Data: Average of duplicate measurement for each concentration (P < 0.001; Tukey's multiple comparison test). **C**. HAase activity (mU/ml) was measured in the serum-free conditioned media of RT4 transfectants treated with or without sHA-F. Data: Mean ± sd; quadruplicate; P < 0.001 for HYAL-1 transfectants. **D**. Immunoblot analysis of serum-free conditioned media of RT4 vector and HYAL-1 transfectants, following 48-hour treatment with sHA-F. **E**. RT4 transfectants were exposed to sHA-F (20 μg/ml) for 72 hours in the growth medium. Following incubation, viable cells were counted. Data: Mean ± sd (quadruplicate); P < 0.0001 for HYAL-1 transfectants.

Since AGF are generated following the degradation of HA by HYAL-1, we treated 253J-L cells with AGF to determine whether the anti-proliferative effects of sHA-F were due to the inhibition of HAase activity. As shown in Figure 2A, AGF addition prevented growth inhibition of 253J-L cells by sHA-F (61% inhibition at 5 μg/ml sHA; no inhibition at 5 μg/ml sHA +AGF). Similar results were obtained in HT1376 and UMUC-3 cells (Supplement Figure 1D and 1E).

### sHA-F induced apoptosis in BCa cells

To examine the mechanism by which sHA-F inhibits cell proliferation we performed cell cycle analysis and apoptosis assays. sHA-F did not induce significant cell cycle arrest in BCa cells. However, sHA-F caused a dose-dependent increase in apoptosis; at IC_50_ for inhibition of cell growth, sHA-F caused a 3-fold induction of apoptosis in both 253J-L and HT1376 cells (Figure 2B). Apoptosis induction by sHA-F was attenuated by the addition of AGF (data not shown). To further establish that the growth inhibitory effects of sHA-F were due to the inhibition of HYAL-1 activity, we overexpressed HYAL-1 in the non-HYAL-1 expressing RT4 cells. HAase activity present in the conditioned media of RT4-HYAL-1 transfectants was comparable to that in the conditioned media of HYAL-1 expressing BCa cells and the activity was inhibited by sHA-F (Figure 2C). sHA-F treatment however, did not alter HYAL-1 protein levels (Figure 2D). HYAL-1 expression increased the proliferation of RT4 cells by 1.4-fold. While sHA-F did not inhibit the growth of vector transfectants, the growth of RT4-HYAL-1 transfectants was inhibited by ∼50% when compared with the untreated control (Figure 2E).

**Figure 3:**
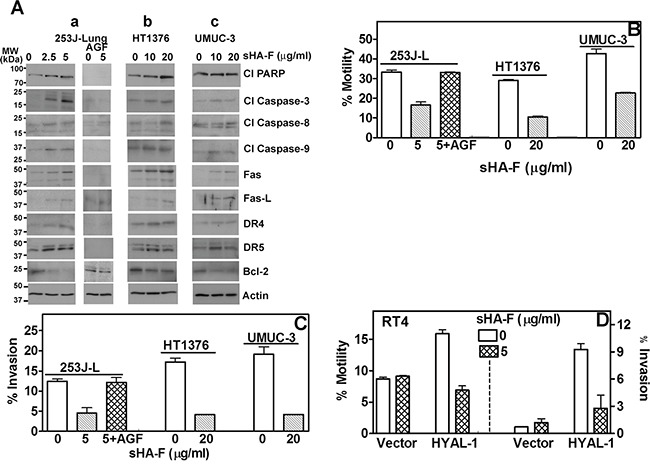
Effect of sHA-F on apoptosis effectors, chemotactic motility and invasion **A**. Immunoblot analysis of BCa cells treated with sHA-F (as indicated) in the presence or absence of AGF for 48 hours. **B–D**. Determination of chemotactic motility and invasion of BCa cells and RT4 transfectants treated with sHA-F and/or AGF. Data Mean ± sd (triplicate; P < 0.001; untreated and sHA-treated BCa cells or transfectants; P > 0.05 between untreated and sHA + AGF treated cells.

In 253J-L, HT1376 and UMUC-3 cells, sHA-F induced the activation of pro-apoptotic effectors (caspase-3, caspase-9, and caspase-8), PARP cleavage and up-regulation of death receptor signaling proteins (Fas, Fas-L, DR4, DR5) by 2-5-fold. Conversely, sHA-F downregulated Bcl-2 in a dose-dependent manner in all three BCa cell lines (Figure 3A). Incubation of 253J-L cells with AGF decreased the basal levels of pro-apoptotic effectors and also prevented their activation by sHA-F treatment (Figure 3A). This suggests that the induction of apoptosis by sHA-F is due to the inhibition of HAase activity.

### sHA-F inhibits chemotactic motility and invasion

Since HYAL-1 promotes tumor invasion and metastasis [7, 9], we investigated whether sHA-F inhibits chemotactic motility and invasiveness of BCa cells and whether AGF addition prevents both activities. As shown in Figure 3B and 3C at IC_50_ of HAase activity inhibition (253J-L: 5-μg/ml; HT1376, UMUC-3: 20-μg/ml), sHA-F caused 50-70% inhibition of chemotactic motility and invasion in BCa cells (P ≤0.0001; unpaired t-test). Furthermore, addition of AGF prevented sHA-F from inhibiting chemotactic motility and invasive activity of 253J-L cells. RT4-HYAL-1 transfectants showed 2- and 10-fold increased chemotactic motility and invasive activity when compared to the vector transfectants, however this increase was neutralized in the presence of sHA-F (Figure 3D). This suggests that inhibition of HAase activity leads to a more indolent phenotype in BCa cells.

### sHA-F downregulates HA-receptor signaling

**Figure 4:**
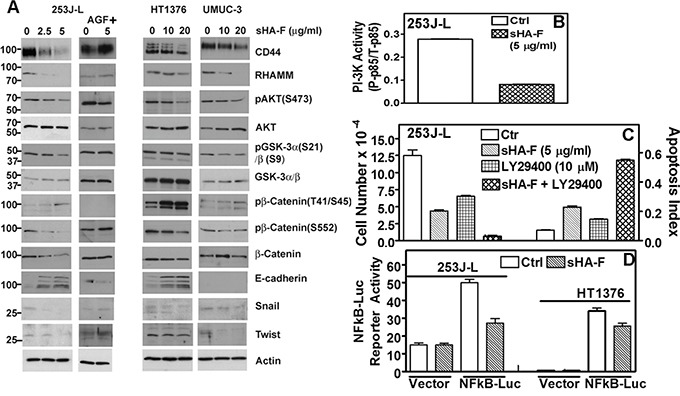
Effect of sHA-F on HA receptors, PI-3K activity and AKT signaling **A**. Immunoblot analysis of HA receptors, AKT signaling effectors and EMT markers in BCa cells treated with sHA-F and/or AGF for 48 hours; β-actin: loading control. Note: While 253J-L and UMUC-3 cells express 90-kDa isoform (standard form) of CD44, HT1376 cells express variant isoforms. **B**. Measurement of PI-3K activity by an ELISA, in 253J-L cells treated with or without sHA –F for 24 hours. Data: Mean ± sd (triplicate); P < 0.0001. **C**. 253J-L cells were treated with sHA-F and/or PI-3K inhibitor LY29400 for 48 hours. Following incubation viable cells were counted and also subjected to apoptosis assay. Data: Mean ± sd (quadruplicate); P < 0.001 (Tukey's test). **D**. 253J-L and HT1376 cells transfected with a vector or pNFκB-luc plasmid were treated with sHA-F. Firefly luciferase and Renilla luciferase activities were assayed after 16 hours. Data: Mean ± sd (triplicate); P < 0.001 for both cell lines.

**Figure 5:**
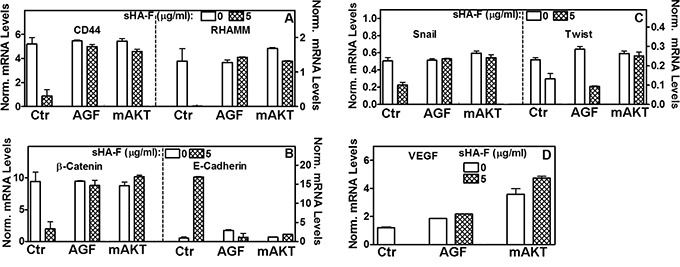
Effect of sHA-F, AGF and mAKT expression on transcript levels 253J-L cells or their mAKT transient transfectants were treated with sHA-F (0, 5-μg/ml) in the presence and/or absence of AGF (50 μg/ml) for 48 hours. Transcript levels of HA receptor, EMT markers and VEGF were measured by RT-q-PCR. Data: Mean ± sd (n = 2 to 5); P<0.001 between control and sHA-F treated cells; P > 0.05 in AGF and mAKT transfectant samples.

We have recently shown that inhibition of HA signaling leads to downregulation of HA receptors, AKT signaling and subsequently of the epithelial mesenchymal transition (EMT) markers. We therefore determined whether by abrogating the generation of angiogenic HA fragments, sHA-F will attenuate HA signaling. As shown in Figure 4A, sHA-F treatment caused a dose-dependent (3-5-fold) decrease in HA-receptors CD44 and RHAMM, pAKT(S473), pGSKα/β, β-Catenin, pβ-Catenin(S552), Snail and Twist protein levels. Contrarily, sHA-F increased E-cadherin (4-6-fold) and pβ-Catenin(T41/S45; 2-3-fold)) levels. sHA-F did not induce E-cadherin expression in UMUC-3 cells, which do not express E-cadherin [34]. Phosphorylation at S552 by AKT induces nuclear localization of β-Catenin, but its phosphorylation at T41/S45 by GSK-3α/β marks β-Catenin for degradation. GSK-3α/β is inactivated by AKT mediated phosphorylation (S21: GSK-3α; S9: GSK-3β). As expected AGF prevented sHA-F from altering the levels of respective proteins or their phosphorylation. sHA-F also significantly downregulated the transcript levels of CD44 (5-fold), RHAMM (100-fold), EMT markers- β-Catenin (5-fold), Snail (2.5-fold), Twist (1.5-fold) -and completely inhibited VEGF expression; however, sHA-F up-regulated the expression of E-cadherin by 10-fold (P<0.001; Figure 5).

### sHA-F inhibits PI-3K activation and AKT signaling

Since sHA-F downregulated pAKT levels, we examined the effect of sHA-F on PI-3K activation. As shown in Figure 4B, in sHA-F treated 253J-L cells, PI-3K activity was downregulated by 3.4-fold (P<0.001). Furthermore, treatment of 253J-L cells with LY29400, a PI-3K inhibitor, and sHA-F synergistically inhibited cell growth by > 95% and induced apoptosis (P<0.001; Figure 4C). Since AKT activates NFkB by inducing degradation of IKB, sHA-F should downregulate the transcriptional activity of NFkB. As shown in Figure 4D, in 253J-L and HT1376 cells 16 hour treatment of sHA-F inhibited NFkB promoter luciferase-reporter activity by ∼40% (P<0.0001).

**Figure 6:**
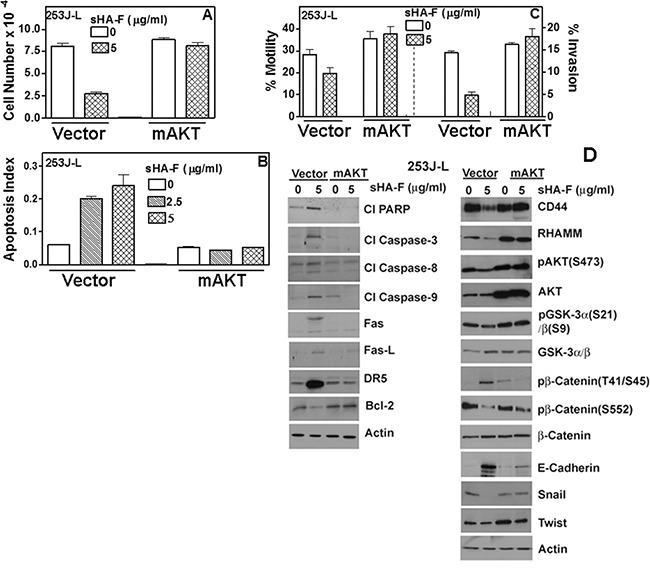
Effect of myr-AKT expression on sHA-F induced effects in 253J-L cells 253J-L cells transiently transfected with vector or myr-AKT constructs were treated with sHA-F for 48 hours and analyzed as in A - D. **A, B, C**. Cell proliferation (A), apoptosis (B) and motility and invasion: (C) Data: Mean ± sd (quadruplicate: A, B; triplicate: C). A, B, C: P < 0.01 in untreated and sHA-F treated samples for vector transfectants; P > 0.05 for mAKT transfectants. **D**. Immunoblot analysis of vector and myr-AKT transfectants; β-actin: loading control.

**Figure 7:**
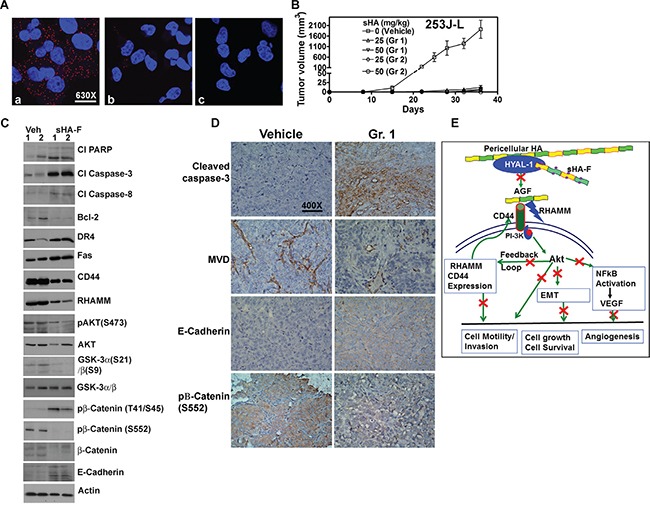
Effect of sHA-F HA signaling and BCa xenograft **A**. PLA for PI-3K and CD44 in 253J-L cells treated with sHA-F (0, 5-μg/ml). Confocal microscopy images at 630X magnification are shown. a and b: cells treated with 0 (a) and 5-μg/ml sHA-F (b); c: IgG control. Note the CD44 and PI-3K complex (red dots) present in untreated cells is significantly reduced in sHA-F treated cells. **B**. Athymic mice were implanted subcutaneously with 253J-L cells and treated with vehicle or sHA-F (25 mg/kg or 50 mg/kg. Group 1: Treatment started on the day of tumor cell injection and continued for two weeks. Group 2: Treatment started on day 9, when tumors became palpable and continued until day 24. **C**. Immunoblot analysis of tumor tissues for apoptosis effectors and HA signaling molecules. β-actin: loading control. **D**. Immunohistochemical analysis. Tumor tissues from the vehicle and sHA-F treatment (Group 1) groups were subjected to activated caspase-3, CD34, E-cadherin and pβ-Catenin(S552) staining using immunohistochemistry as described in the Supplemental information. Magnification: 400X. **E**. Proposed model for the mechanism of action for sHA-F.

Since AKT signaling was downregulated by sHA-F, we investigated whether sHA-F effects could be attenuated by overexpression of myristoylated–AKT (mAKT), which constitutively activates AKT signaling. In mAKT transfectants, sHA-F treatment neither inhibited growth nor induced apoptosis (Figure 6A, 6B). mAKT expression caused a slight increase in chemotactic motility (1.3-fold) and invasion (1.2-fold) in 253J-L cells; however, sHA-F treatment did not inhibit either of these activities (Figure 6C). RT-q-PCR analysis showed that mAKT overexpression attenuated sHA-F induced downregulation of CD44, RHAMM, β-Catenin, Snail, Twist and VEGF, and up-regulation of E-Cadherin (Figure 5). Immunoblot analysis showed that mAKT overexpression attenuated the up-regulation of apoptosis effectors (i.e., activation of caspases, PARP cleavage, Fas, Fas-L, DR5) and the downregulation of HA receptors, AKT signaling (pAKT, Bcl-2, pGSK-3α/β, pβ-Cateinin(S552) and EMT markers (β-Cateinin, Snail, Twist; Figure 6D). In addition, mAKT overexpression inhibited up-regulation of E-cadherin, and pβ-Catenin(T41/S45). This suggests that inhibition of AKT activation due to the blockade of AGF-induced signaling is the major target of sHA-F. To determine how sHA-F causes downregulation of PI-3K/AKT signaling, we examined whether sHA-F decreases a complex formation between HA receptors and PI-3K [35, 36]. PLA followed by confocal microscopy showed that in 253J-L cells, CD44 and PI-3K were present in the same microdomains (proximity < 40 nm); however, the complex formation was inhibited > 90% following sHA-F treatment (Figure 7A).

### Effect of sHA-F on tumor growth

To determine whether sHA-F has chemopreventive and/or therapeutic effects, in the 253J-L xenograft model, two-week treatment of sHA-F was started either on the day of tumor cell injection (Group 1) or after the tumors became palpable (i.e., day 9; Group 2). As shown in Figure 7B, at both 25 and 50-mg/kg doses, sHA-F significantly inhibited tumor growth in both groups. The differences in animal weights in the treatment and vehicle groups were not statistically significant (Supplement Figure 2A); we have previously shown that sHA-derivatives do not cause serum or organ toxicity [10]. On day 36, the average tumor weights in the vehicle group (0.87 ±0.17 g) were significantly higher than in the sHA-F treatment groups: 25-mg/kg (Group 1: 0.09 ±0.09 g; Group 2: 0.08 ±0.1 g) and sHA-F 50-mg/kg (Group 1: 0.06 ±0.14 g; Group 2: 0.01 ±0.02 g); P <0.001 (Tukey's multiple comparison test; Supplement Figure 2B). When compared to the tumor tissues from the vehicle treated group, tumor tissues from the 25-mg/kg sHA-F treatment group (i.e. Group 1) showed increased levels of cleaved PARP, activated caspase – 3, 8, DR4, Fas, E-cadherin and pβ-Cateinin(T41/S45), but a decrease in Bcl-2, pAKT, pGSK-3α/β, β-Cateinin, pβ-Cateinin(S552) levels (Figure 7C). Immunohistochemistry on tumor tissues in animals from the vehicle group and Group 1 (25-mg/kg) confirmed the downregulation of pβ-Catenin(S552) and the up-regulation of cleaved caspase-3 and E-cadherin in the sHA-F treated group (Figure 7D). Furthermore, MVD was significantly reduced in tumor tissues in Group 1 animals when compared to the vehicle treated group (MVD ± sd: vehicle: 13.1 ± 3.6; Group 1: 1.7 ± 2.1; P<0.0001; Figure 7D). Tissues from 50-mg/kg treatment group could not be analyzed because tumor growth was nearly abrogated, leaving little tissue for analysis. Nevertheless, inhibition of tumor growth by sHA-F appears to be due to induction of apoptosis and inhibition of AKT signaling. To determine whether sHA-F treatment can inhibit the growth of an established tumor, in the HT1376 xenograft model, sHA-F treatment was started after tumors reached ∼ 100 mm^3^. In this model, sHA-F treatment (50-mg/kg) also significantly inhibited tumor growth (vehicle: 1054±331.3 mm^3^ at day 40; sHA-F: 293.2±198.6 mm^3^ at day 48), without affecting animal weight (Supplement Figure 2C - 2E).

## DISCUSSION

BCa is the first cancer model system in which HYAL-1 was found to be a molecular determinant of tumor growth, invasion, and angiogenesis and also as a diagnostic and prognostic marker [2, 7, 11, 12, 14, 15, 17, 37]. The present study should significantly advance the field, as it shows targeting of HYAL-1 activity with specific inhibitors can be a potential therapeutic avenue for BCa patients. The impact of this study is the demonstration that sulfation of AGF, which are produced when HA is degraded by HYAL-1, generates a potent inhibitor of HAase activity that has significant antitumor activity but little toxicity. Furthermore the antitumor activity of sHA-F is largely, if not exclusively due to the inhibition of HYAL-1 activity. Since the tumor-associated HA-HAase system is active in a variety of carcinomas, the broader implication of this study would be that targeting of HYAL-1 with small molecule uncompetitive inhibitors could be a potential avenue for cancer therapy.

Inhibition of HYAL-1 activity as the sole target of sHA-F induced antitumor activity is based on our results that: 1. sHA-F was inhibitory only to HYAL-1 expressing cells; 2. Inhibitory effects of sHA-F on growth, motility, invasion and intracellular signaling were attenuated by AGF, which are the “signaling fragments” generated by HYAL-1 mediated HA degradation; 3. Expression of HYAL-1 in a non-invasive BCa cell line increased proliferation, motility and invasion but also sensitized the cells to sHA-F treatment. 4. sHA-F inhibited tumor growth in HYAL-1 expressing xenograft models.

Although HYAL-1 expressing BCa cells are susceptible to growth inhibition by sHA-F, they differ in terms of their sensitivity to sHA-F. For example, 253J-L cells were more sensitive to sHA-F than HT1376 and UMUC-3 cells; the IC_50_ for sHA-F induced inhibition of phenotypic properties was 4-fold lower in 253J-L cells than the IC_50_ for HT1376 and UMUC-3 cells. The reason why 253J-L cells are more sensitive to sHA-F treatment than HT1376 and UMUC-3 cells is plausibly related to the balance of tumor-associated HA-HAase system and the cellular dependence on the HA-HYAL-1 axis. For example, HT1376 cells produce ∼ 1.5-3-fold more HA and HYAL-1 than 253J-L cells ([4]; Figure 1 and Supplemental Figure 1). Therefore, these cells may require higher concentration of sHA-F for the inhibition of endogenous HAase activity and phenotypic readouts. Contrarily, UMUC-3 cells produce similar amounts of HA but lower amounts of HYAL-1 ([4] and Supplemental Figure 1). It is possible that these cells are “less addicted” to the HA-HAase pathway.

Tumor associated HA-HYAL-1 system has been shown to activate AKT and downstream signaling, which includes NFKB activation, VEGF production, and EMT, via β-catenin activation [38–41]. Although sHA-F abrogates the generation of AGF, pericellular HA should still be able to induce HA signaling through HA receptors. However, the fact that HA signaling was blocked in sHA-F treated cells and xenograft models raise two possibilities; the first is that in tumor tissues AGF are the primary signaling molecules. This possibility is corroborated by our observations that AGF are able to attenuate sHA effects on phenotypic readouts and HA signaling. It has been shown that high molecular mass HA is inhibitory to cancer growth [42, 43]. Second and perhaps more likely, is that due to a feedback loop, inhibition of AKT activation leads to downregulation of HA receptors, which should abrogate all HA signaling. Based on these mechanistic studies, we propose a model for sHA-F action (Figure 7E). HYAL-1 degrades pericellular HA to generate AGF, which then bind to HA receptors CD44 and RHAMM and activate the PI-3K/AKT signaling pathway. AKT activation induces cell growth, cell survival, motility/invasion, and promotes EMT and NFkB activation/VEGF expression. AKT activation also induces CD44 and RHAMM expression and thus, drives the signaling pathway. By blocking AGF production, sHA-F inhibits the first step in this signaling cascade.

We have previously shown that silencing of HYAL-1 expression in the HT1376 xenograft model leads to the inhibition of tumor growth, invasion and angiogenesis. Furthermore, these tumors resemble benign neoplasia [7]. Consistent with its effects on HAase activity, sHA-F treatment significantly inhibited growth of 253J-L and HT1376 tumors. This inhibition was observed regardless of whether the treatment commenced on the day of tumor cell implantation, or when the tumors were established. The study's findings that in tumor tissues from sHA-F treated animals, there was inhibition of AKT activation, EMT effectors and angiogenesis but an induction of E-cadherin and caspase-3, confirm that the antitumor activity of sHA-F *in vivo* is due to the inhibition of HAase activity. We have previously shown that sHA/derivatives do not display serum or organ toxicity and have a stable serum half-life [10].

Based on the phenotypic readouts, mechanistic studies and activity of sHA-F in two xenograft models, our study demonstrates that tumor-derived HAase, HYAL-1 can be targeted to control BCa growth and progression. The study's findings suggest that sHA-F may be useful as an intravesical agent to reduce BCa recurrence, and/or adjuvant setting to control its malignant progression.

## MATERIALS AND METHODS

### Cells

BCa cell lines - HT1376, 5637, TCC-SUP, T24, RT4, and UMUC-3, and immortalized normal urothelial cells SV-HUC1 were purchased from American Type Culture Collection. 253J-Lung cells were provided by Dr. Colin Dinney (MD Anderson Cancer Center). Immortalized normal bladder epithelial cell line Urotsa was provided by Dr. Donald Sens, University of North Dakota. BCa cells were authenticated by Genetica DNA Laboratories Inc., Cincinnati OH. BCa and Urotsa cells were cultured in RPMI 1640 + 10% fetal bovine serum and gentamicin (growth medium). SV-HUC1 cells were cultured in F12K medium + 10% fetal bovine serum. All experiments were conducted between passages 2 and10.

### Angiogenic HA fragment (AGF) and sHA-F

HA fragments of average molecular mass 12,000 Dalton (8,000 – 15,000; AGF) and of average molecular mass 2,000 Dalton were kindly provided by Genzyme Corporation. Tributylamine salt of HA fragments was sulfated using SO3^−^ pyridine, as described before [32]. Antibodies, constructs and reagents used in this study are described in the Supplemental Information.

### HAase activity ELISA-like assay

BCa cells (70% confluent cultures) were exposed to sHA-F (0 – 40-μg/ml) in RPMI 1640 supplemented with insulin, transferrin and selenium (serum-free RPMI) for 24 hours. Conditioned media were subjected to HAase activity ELISA-like assay, as described before [7]. HAase activity (mU/ml) was normalized to cell number or to total protein concentration (mg/ml). In some cases, conditioned media were incubated in the presence or absence of sHA-F at 4° C for 1 hour prior to adding to the ELISA wells. For immunoblot analysis of HYAL-1 protein in the conditioned medium, normalization was also performed using cell number, and confirmed using actin as a loading control.

### Cell proliferation and apoptosis assays

BCa and normal urothelial cells (1.5×10^4^ cells/well) cultured in growth medium were exposed to sHA-F (0 – 40-μg/ml) either alone or in the presence of AGF (50-μg/ml) or a PI3-kinase inhibitor LY29400 (0, 10-μM) for 48 to 72 hours. Following incubation, viable cells were counted (Trypan blue staining). For apoptosis assay, cells were treated for 48 hours and apoptosis was measured using the Cell Death ELISA Plus kit (Roche Diagnostics; Indianapolis, as per the manufacturer's instruction; the results were expressed as apoptosis index (per 5,000 cells). Apoptosis index: optical density measurement at 405 nm (reference wavelength 490 nm) and subtraction of the negative control readings.

### Motility and invasion assays

Matrigel™ invasion and motility assays were carried out as described previously [7, 27] except that sHA-F and/or AGF were added in both chambers of the Transwell (Supplemental Information). Incubation times for motility and invasion assays were 18 and 48 hours, respectively.

### Immunoblot and phosphoinositide 3-kinase (PI-3K) assays

BCa cells (∼ 50,000 cells/6-well plate) were exposed to sHA-F (0 - 20-μg/ml) for 48 hours. In some wells, 50-μg/ml AGF was added at the time of sHA-F addition. Tumor tissue extracts from vehicle and treated animals were prepared as described before [26, 27]. Cell lysates (∼20,000 cell equivalent) and tissue extracts were analyzed by immunoblotting using specific antibodies; β-actin was used as a loading control. 253J-L cells treated with sHA-F (0 or 5-μg/ml) for 24 hours were subjected to PI-3K activity assay using a PI-3K p85 colorimetric ELISA kit (Active Motif; Carlsbad, CA).

### Reverse transcription quantitative polymerase chain reaction (RT-qPCR)

Total RNA isolated from BCa cells was subjected to Q-PCR using the Ssofast Evagreen Supermix (BioRad, Hercules, CA) and gene specific primers described in Supplement Table 1. mRNA levels were normalized to β-actin mRNA levels, and the normalized transcript levels for each gene were calculated as (1/2^Δct^ x 100); ΔCt = Cq (Transcript) - Cq (β-actin).

### Transfectants

RT4 cells were transfected with a HYAL-1 construct and the transfectants were selected in Geneticin (Invitrogen, Carlsbad, CA), as described before [7]. 253J-L cells were transiently transfected with Myr-HA-AKT1 plasmid (mAKT; Addgene, Inc., Cambridge, MA), or vector alone. Twenty-four hours following transfection, cells were exposed to sHA-F (5-μg/ml) for 48 hours and then analyzed for proliferation, apoptosis, invasion, and protein expression; motility and RT-q-PCR assays were performed following 18 and 24-hour exposure, respectively. For NFκB-reporter assays, 253J-L and HT1376 cells were transiently transfected with a pNFκB-luc, pGL4.10[luc2] vector (Promega Corp.; Madison WI) or Renilla-luc plasmid, and 8 hours following transfection, the cells were exposed to sHA-F (5-μg/ml). Firefly luciferase reporter activity was measured 16 hours later. Promoter activity was normalized to Renilla luciferase activity [10].

### Proximal ligation assay (PLA)

253J-L cells exposed to sHA-F (5-μg/ml) for 24 hours were fixed in paraformaldehyde (4%) and permeabilized using 0.25% Triton X-100. Permeabilized cells were subjected to PLA using the Duolink ®*In situ* reagents (Sigma Aldrich) as per the manufacturer's protocol, that involved incubation of the permeabilized cells with rabbit anti-PI-3K p85 (1:650) and mouse anti-CD44 (1:650) antibodies at 37°C for 1 hour. The slides were observed under a Zeiss LSM700 Confocal microscope equipped with multi-variant fluorescence filters in two channels (red and blue) under a 63X oil-immersion objective lens, as described before [33].

### Tumor xenograft models

253J-L cell suspension (2×10^6^ cells/0.1 ml) was mixed 1:1 with Matrigel™ and implanted subcutaneously on the dorsal flank of 5-6 week old athymic mice. The mice were randomly divided into five groups and injected intraperitoneally twice weekly with either phosphate buffered saline (PBS) or sHA-F as follows: Vehicle (n = 8); Gr. 1: 25-mg/kg (n = 7) and 50-mg/kg (n = 9) sHA-F treatment, starting on day 1 for two weeks; Gr. 2: 25-mg/kg (n = 5) and 50-mg/kg (n = 5) sHA-F treatment, starting on day 9 (when tumors became palpable) and terminating on day 24. Immunohistochemical localization of microvessels, activated caspase-3, E-cadherin and pβ-catenin(S552) was performed on tumor specimens from vehicle and treatment (sHA-F 25 mg/kg Group 1) as described in the Supplemental Information [10, 26, 27]. Microvessel density (MVD) was determined by counting the microvessels in high power fields (400X magnification). Tumor tissue extracts were prepared and subjected to immunoblotting as described before [26, 27]. In the HT1376 xenograft model, tumor cells (1×10^6^ cells) were implanted subcutaneously on the dorsal flank of 5-6 week old athymic mice. When tumor size reached ∼ 100 mm^3^, the mice were treated with vehicle or sHA-F (50 mg/kg), as described above for 40 days (vehicle) or 48 days (sHA-F group). Tumor volume, animal weight and tumor weight at euthanasia were measured as described before [26, 27].

### Statistical analysis

Mean ± sd was computed for quantifiable parameters (e.g., cell number, apoptosis index, % motility, % invasion, tumor volume). Differences among vehicle/control and treatment groups were compared by one-way ANOVA followed by either unpaired t-test (e.g., control versus treatment) or Tukey's multiple comparison test when comparing more than two groups (e.g. vector control, vector sHA-F, mAKT control, mAKT sHA-F). All P-values were two-tailed. Statistical analyses were performed using the GraphPad Prism software.

## SUPPLEMENTARY MATERIALS FIGURES AND TABLES



## Abbreviations

AGF: angiogenic hyaluronic acid fragments
HA: hyaluronic acid
HAase: hyaluronidase
myr-AKT: myristoylated AKT
sHA: sulfated hyaluronic acid
sHA-F: sulfated hyaluronic acid fragment(s)
RT-qPCR: Quantitative reverse transcription PCR.
