# Supplementary material for: Antitumor activity of sulfated hyaluronic acid fragments in pre-clinical models of bladder cancer
Source: Oncotarget. 2016 Jul 11;8(15):24262–74. doi: 10.18632/oncotarget.10529 (PMC5421845; doi:10.18632/oncotarget.10529)
Supplement: Supplementary file 1 [file oncotarget-08-24262-s001.pdf]

Antitumor activity of sulfated hyaluronic acid fragments in pre-clinical models of bladder cancer

SUPPLEMENTARY DATA

MATERIALS

Antibodies and reagents

BD Bioscience: Bcl-2 (4D7, Mouse Monoclonal); Cell Signaling, Danvers MA: pAkt S473 (EP2109Y; rabbit Monoclonal), Akt1 (C73H10; rabbit monoclonal), E-Cadherin (24E10; rabbit monoclonal), pβ-Catenin(S552; rabbit polyclonal # 9566); pβ-Catenin (T41/S45; rabbit polyclonal); glycogen synthase kinase 3 (GSK-3 α (rabbit polyclonal # 9338) and β (rabbit monoclonal 27C10) - a cocktail was prepared for simultaneous detection by immunoblotting), pGSK-3 α/β (S21/S9; rabbit polyclonal); Snail (L70G2, mouse monoclonal); Cleaved Caspase-8 (Asp691, 18C8, rabbit monoclonal); Cleaved Caspase-9 (Asp330, rabbit polyclonal); cleaved PARP (Asp214, D64E10, rabbit monoclonal); Santa Cruz Biotechnology Inc. (Santa Cruz, CA): CD44 (DF1485, Rabbit Monoclonal); Fas (DX2, mouse monoclonal); Fas-L (C-178, rabbit polyclonal); Twist (H-81, rabbit polyclonal) Epitomics/Abcam – Cambridge, MA: caspase-3 (active, E83-77 rabbit monoclonal); β-catenin (E247, rabbit monoclonal); Novocastra Leica Microsystems (Bannockburn, IL): RHAMM (CD168, H-90 mouse monoclonal); Novus Biologicals, Littleton, CO.; EMD Millipore USA: Hyaluronic Acid Binding Protein, Bovine Nasal Cartilage, Biotinylated; Invitrogen, Carlsbad, CA: Lipofectamine® 2000; Addgene, Inc., Cambridge, MA: Myr-HA-AKT1 plasmid (myr-AKT;). Rabbit anti-HYAL-1 antibody used in this study has been described before [10]. All antibodies were used at dilutions between 1:500 and 1:1000.

Reagents and kits

LY29400: Cayman Chemical Company, Michigan; Cell Death ELISA Plus kit: Roche Diagnostics; Pleasanton, CA; Myr-HA-Akt1 plasmid: Addgene Inc., Cambridge; RNeasy Mini Kit (QIAGEN); iScript cDNA synthesis kit (BioRad #170-8890); SsoFast EvaGreen Supermix (BioRad #172-5200).

DETAILED METHODS

Detailed description of motility and invasion assays

Matrigel™ invasion assay was carried out as described previously [13] except that sHA-F was added in both chambers of the Transwell but not in Matrigel™. sHA-F concentrations were as follows: 253J-L: 5-µg/ml; HT1376 and UMUC-3: 20- µg/ml. The cells in the top chamber were cultured in RPMI 1640 + ITS (insulin, transferrin and selenium, Sigma Aldrich, St Luis, MO) medium and the bottom chamber contained growth medium as the chemoattractant. Invasion was assayed after 48 hour incubation. For motility assay, 8-µm pore Transwell with similar experimental set up was used and the migration of the cells was assayed after 18 hour incubation. In both assays, AGF (50-µg/ml) was added to the top and the bottom chambers of some wells. Cell densities in the top chamber and the cells adhered to the bottom of the filter were determined using MTT (3-(4,5-Dimethylthiazol-2-yl)-2,5-diphenyltetrazolium bromide, a yellow tetrazole), assay. Optical density was measured at 575 nm with correction at 650 nm. To neutralize the effect of sHA on cell growth, percent invasion or motility were calculated as (O.D. bottom chamber ÷ O.D. (top + bottom chambers)) x100.

Detailed description of transient transfection assays with myr-AKT plasmid

253J-L cells (50,000 cells/12-well plates) were transiently transfected with myr-HA-AKT plasmid (Addgene, Inc., Cambridge, MA) using Lipofectamine 2000 (Invitrogen, Carlsbad, CA). Twenty-four hour following transfection, cells were trypsinized, counted and plated for cell proliferation, apoptosis, motility, invasion assays or gene and protein expression analyses. During incubation cells were exposed to 0 or 5-µg/ml sHA-F.

Immunohistochemistry

For immunohistochemistry, paraffin fixed tissue sections (5-µM) were stained microvessels (anti-CD34 1:20 dilution), activated Caspase-3 (1:50 dilution), E-cadherin (anti-E-cadherin 1: 1,000) and pβ-CateninS552 (anti- pβ-Catenin S552, 1:1,000), using the IHC procedure described before. Microvessel density (MVD) was determined by counting microvessels and activated caspase-3 positive cells, respectively using a Nikon H550L microscope with a video screen camera, under 400X magnification.

SUPPLEMENTARY FIGURES AND TABLE

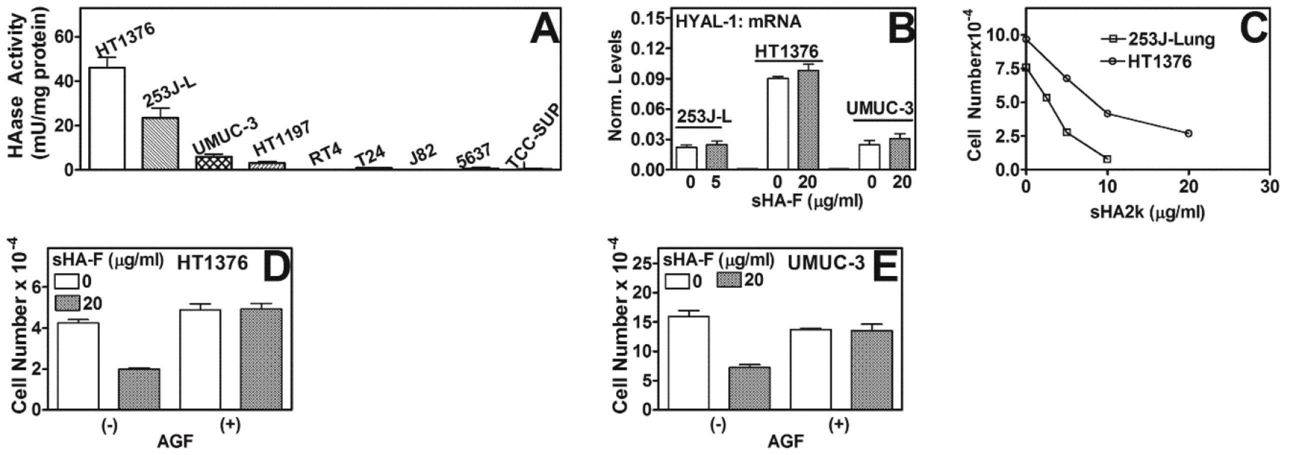

Supplementary Figure S1: Effect of sHA-F *in vitro* and in xenograft. A. HAase activity was measured in the serum-free conditioned media of BCa cell lines using a HAase ELISA-like assay. The activity (mU/ml) was normalized to total protein concentration (mg/ml). Data: average of duplicate determination. **B.** 253J-L, HT1376 and UMUC-3 cells were exposed to sHA-F for 48 hours. Following incubation, HYAL-1 mRNA levels were measured and normalized to β-actin mRNA levels. Data: Mean ± sd (quadruplicate). **C.** 253J-Lung and HT1376 cells were treated with sHA2k and 72 hour later viable cells were counted. Data: Mean ± sd (quadruplicate). **D** and **E.** HT1376 (D) and UMUC-3 (E) cells were treated with sHA-F in the presence or absence of AGF (50-µg/ml) for 48 hours and viable cells were counted. Data: Mean ± sd; quadruplicate.

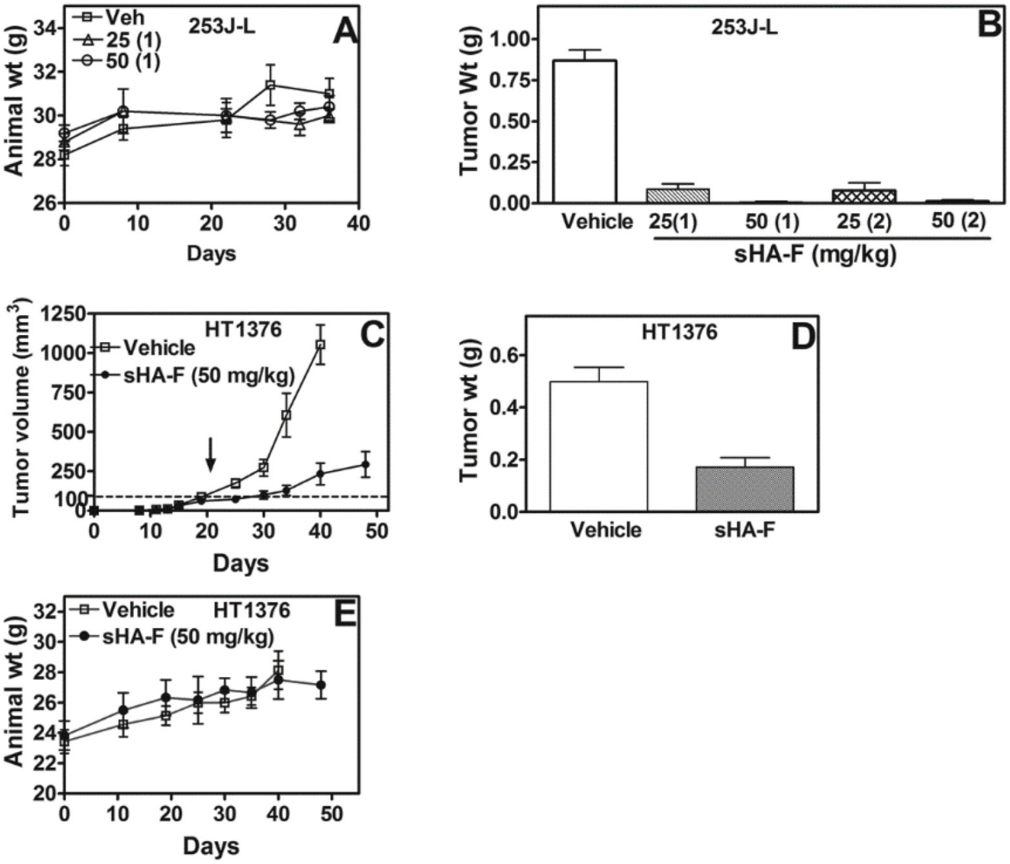

Supplementary Figure S2: Effect of sHA-F in xenograft models. A. Weight of animals in the vehicle and treatment groups of 253J-L xenograft, as described under Figure 7B. Note: The increased animal weight in the vehicle group is due to tumor burden. Data: Mean ± sd. **B. Tumor weight:** 253J-Ltumors from each group (vehicle and treatment) were collected at necropsy and weighed. Data: Mean ± sd. **C–E.** Athymic mice were implanted subcutaneously with HT1376 cells. Mice were injected intraperitoneally twice weekly with either phosphate buffered saline (vehicle) or sHA-F (50 mg/kg) once the tumor volume reached ~ 100 mm^3. C: Tumor volume; D: Tumor weight; vehicle group, at day 40; sHA-F group at day 48. E: Weight of animals in the vehicle and treatment groups. Data: Mean ± sd.

Supplementary Table S1: Polymerase chain reaction (PCR) primers

Gene	Forward Primer	Reverse Primer
CD44s	CTGTACACCCCATCCCAGAC	TGTGTCTTGGTCTCTGGTAGC
CD44v	CAGGTGGAAGAAGAGACC CAA	GCTGAGGTCACTGGGATG AA
RHAMM	CAGCTGGAAGATGAAGAAGGA	GCATGTAGTTGTAGCTGAAAAGG
β-Catenin	ATGGAACCAGACAGAAAAGC	GCTACTTGTTCTTGAGTGAAG
E-Cadherin	ATGCTGAGGATGATTGAGGTGGGT	CAAATGTGTTCAGCTCAGCCAGCA
Snail	GAGGCGGTGGCAGACTAG	GACACATCGGTCAGACCAG
Twist	GCGGGAGTCCGCAGTCTTA	TGAATCTTGCTCAGCTTGTC
VEGF	GCACCCATGGCAGAAGG	CTCGATTGGATGGCAGTAGCT

List of primers used for performing reverse transcription quantitative PCR for various human genes.
